# Prevention of postpartum haemorrhage: a distributional approach for analysis

**DOI:** 10.1186/s12978-018-0530-7

**Published:** 2018-06-22

**Authors:** Gilda Piaggio, José Ferreira de Carvalho, Fernando Althabe

**Affiliations:** 1 Statistika Consultoria, Campinas, São Paulo Brazil; 2 Statistika Consultoria, Campinas, São Paulo Brazil; 3 Institute for Clinical Effectiveness and Health Policy (IECS-CONICET), Department of Mother & Child Health Research, Buenos Aires, Argentina

**Keywords:** Postpartum blood loss, Postpartum haemorrhage, Severe postpartum haemorrhage, Lognormal distribution

## Abstract

**Background:**

There is empirical evidence that measured postpartum blood loss has a lognormal distribution. This feature can be used to analyze events of the type ‘blood loss greater than a certain cutoff point’ using a lognormal approach, which takes into account all the quantitative observations, as opposed to dichotomizing the variable blood loss volume into two categories. This lognormal approach uses all the information contained in the data and is expected to provide more efficient estimates of proportions and relative risk when comparing treatments to prevent postpartum haemorrhage. As a consequence, sample size can be reduced in clinical trials, while keeping the statistical precision requirements.

**Methods:**

The authors illustrate how a lognormal approach can be used in this situation, using data from a clinical trial and the event ‘blood loss greater than 1000 mL’.

**Results:**

Estimates of the proportions of this event for each treatment, and relative risks obtained with this method are presented and compared with the standard estimates obtained by dichotomizing measured blood loss volume. An example of how the blood loss distributions of two treatments can be compared is also presented. Different scenarios of the sample size needed to compare two treatments or interventions are presented to illustrate how with the lognormal approach the size of a clinical trial can be reduced.

**Conclusions:**

A distributional approach for postpartum blood loss using the lognormal distribution fitted to the data results in more precise estimates of risks of events and relative risks, compared to the use of binomial proportions of events. It also results in reduced required sample size for clinical trials.

**Trial registration:**

This paper reports a secondary analysis for a trial that was registered at clinicaltrials.gov (NCT00781066).

## Background

The development of an adequate statistical analysis technique to analyze a continuous variable depends on the knowledge of its distribution. Many variables in biology and medicine follow the normal distribution and standard statistical techniques can be applied to compare means. However, when the distribution is not normal, the standard statistical techniques are no longer appropriate and a transformation is often used to normalize the distribution. This has been the case with postpartum blood loss, for which a logarithmic transformation has been used to compare medians, [1] based on the observation that the blood loss distribution is positively skewed, as the lognormal distribution. Also, there are oftentimes physical or biological justifications for a variable to have a specific distribution. The lognormal distribution is a result of many independent small multiplicative effects. It is a simple model that applies to many problems such as body and tumor mass (weight) [2], and blood pressure [3]. Examination of simple histograms of blood loss reveals right skewed distributions that resemble the lognormal distribution, and more in-depth and formal statistical analysis showed that indeed the lognormal distribution fits the blood loss distribution very well. [4]

In the case of postpartum blood loss, there is interest to compare events of the type ‘blood loss beyond a certain cutoff point’ because the loss of large amounts of blood postpartum can lead to severe maternal morbidity and mortality [5, 6]. Therefore there has been a concern to find efficient treatments or interventions to prevent postpartum haemorrhage (PPH), defined as blood loss of 500 ml or more within 24 h after birth, and severe PPH (sPPH) as blood loss of 1000 ml or more within 24 h after birth [7].

Measured blood loss is thus categorized in two categories, by means of an indicator variable of blood loss greater than a certain cutoff point. The estimation approach used so far has been to compute the sample proportion of women with blood loss equal to or above the cutoff point, or a binomial proportion. However, the categorization of a dependent variable results in a loss of power to detect true effects, which is substantial if the distribution is highly skewed and if the categorization is done in few categories, or both [8].

We have shown empirically, elsewhere [4], that the distribution of postpartum blood loss volume is lognormal, using data provided by the authors from three trials that compared two drugs [1, 9] or two management procedures for the third stage of labour [10], and one observational study [11]. We used this finding to propose an analysis approach based on the lognormal distribution, resulting in more efficient estimates of proportions and relative risk and in a reduction of the sample size needed in clinical trials that compare proportions between treatments [4]. In this paper we illustrate how this approach (denoted ‘the lognormal approach’) can be used to analyze data from one of these trials, the Althabe et al. trial [1].

## Methods

Descriptive histograms by treatment are constructed to have a first view of the distributions.

The lognormal approach that we propose uses measured blood loss observations without categorizing this variable. It consists of the following steps:

The procedure starts by fitting a three-parameter lognormal distribution [12] to the data. The parameters of the lognormal distribution are estimated by maximum likelihood.

Goodness of fit is assessed using probabilistic plots, consisting in plotting the quantiles of the fitted lognormal distribution against the observed blood loss values. If the fit is good, then the points will fall on a straight line. The probabilistic plot is also used to detect presence of outliers and to assess the quality of the data.

Once a lognormal distribution is considered to be a good fit to the data, the fit is visualized by plotting the cumulative distribution function for the observed data (‘empirical cumulative distribution function’) together with the fitted lognormal cumulative distribution function, or, alternatively, its complement, denoted here as the survival function. The “survival” function gives the probability of having blood loss MORE than a particular value. It is R(v) = 1-F(v), where F(v) is the cumulative probability function.

The probability of an event of the type ‘blood loss greater than a cutoff point’ is just the survival function at the cutoff point. For example, the proportion of sPPH is just the survival distribution at the point 1000.

Comparison of proportions between treatments and computation of relative risks with confidence intervals are done using bootstrap techniques. We generated one thousand bootstrap samples for each treatment. The estimates of the proportions of sPPH and PPH are then computed for each bootstrap sample, for each treatment. [13] The two bootstrap samples tables are matched by row (sample) and the relative risks computed. From the distribution of the 1000 bootstrapped relative risks, the 95% confidence interval can be obtained from the 2.5 and 97.5% percentiles of the distribution of the 1000 samples.

To illustrate the gain in precision for this data, we estimated the proportions and relative risk in the standard way, with 95% confidence intervals, denoted the binomial approach. For the proportions, we calculated the width of the 95% confidence intervals for both approaches, the lognormal approach and the binomial approach, and calculated their ratio as a quantification of the gain in precision. For the relative risk we applied a similar procedure but on the relative scale.

We also illustrate tests of hypothesis using the two approaches. For the test of equality of the sPPH proportions between the two treatments, using the binomial approach, we report the Pearson chi-square statistic and *p*-value. To test the equality of the distributions for the two treatments using the lognormal fits, we proceed sequentially: first we test the model with equal scale parameters versus the full model (both location and scale parameters possibly different). If the null hypothesis with equal scale parameters is not rejected, we test the model with equal location parameters against the model with possible different location parameters and conclude about whether the distributions differ at, say, 5% level of significance.

All the computations for fitting distributions and obtaining estimates were done with JMP® 13 software. [14]

Computations for sample size calculations were done with SAS® software version 9.4 (PROC POWER procedure) [15].

## Results

### Descriptive histograms of blood loss volume

**Fig. 1:**
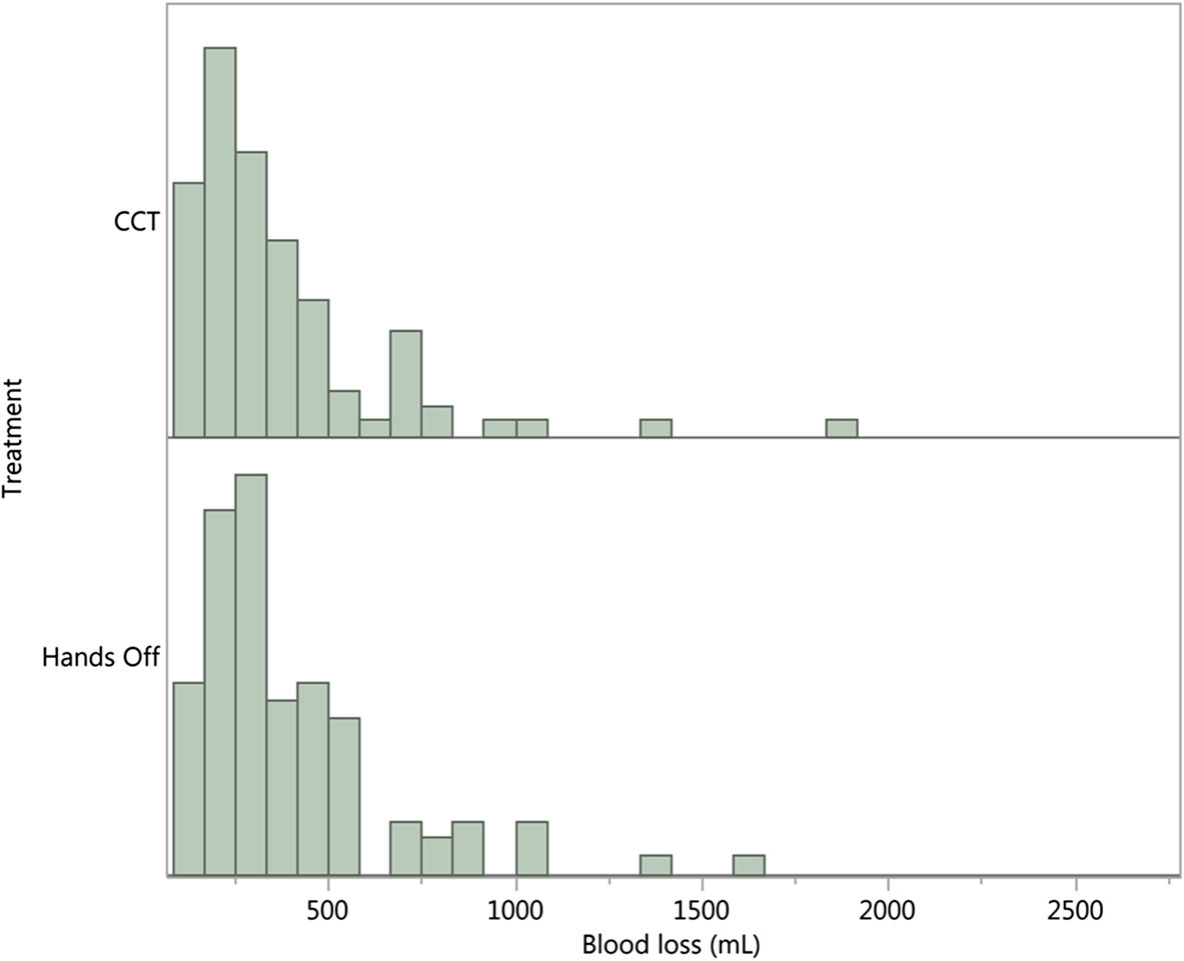
Frequency distribution of blood loss volume (mL) by treatment, Althabe et al. trial [1]

In Fig. 1 we show histograms of frequency distributions of blood loss for the Althabe et al. trial (1), by treatment. The distributions are right-skewed, but from the histograms we cannot specify the statistical distribution originating the data.

### Fitting a lognormal distribution

A three-parameter lognormal distribution (threshold lognormal, abbreviated as THLN) was fitted to the data by maximum likelihood. The third parameter was added because it improved the fit compared to the two-parameter lognormal distribution. The estimated parameters are shown in Table 1, with their standard errors and the 95% confidence intervals.

**Table 1:** Estimated parameters for the fitted threshold lognormal distribution for the Althabe et al. trial [1] (the three parameters are denoted location, scale and threshold parameters); treatments are hands-off and control cord traction (CCT)

Treatment	Parameter	Estimate	Std Error	95% CI
Hands-off	location	5.57	0.141	5.30 to 5.85
	scale	0.72	0.101	0.52 to 0.92
	threshold	55.14	24.474	7.18 to 103.11
CCT	location	5.37	0.132	5.11 to 5.63
	scale	0.80	0.101	0.60 to 1.00
	threshold	62.88	16.414	30.71 to 95.05

**Fig. 2:**
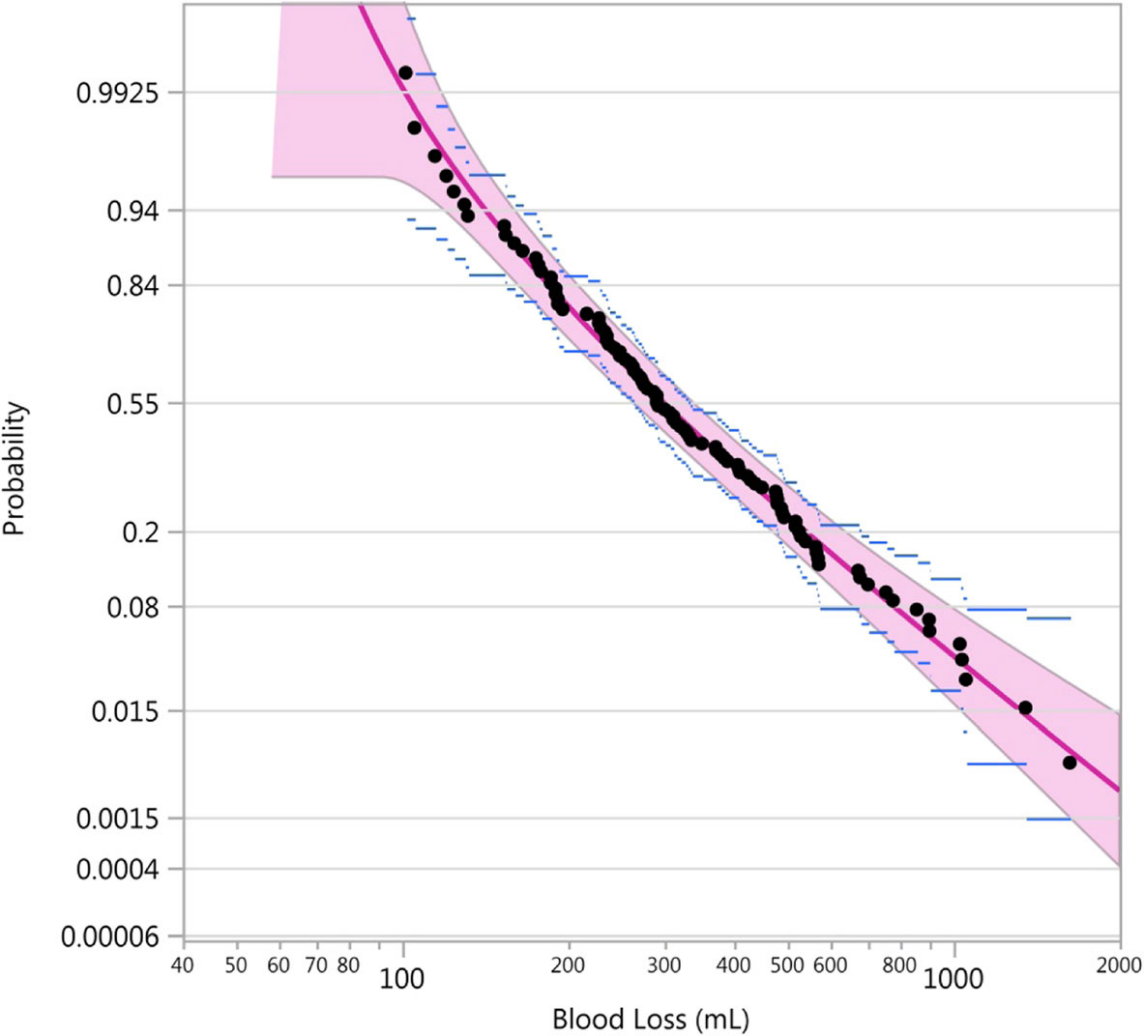
Probabilistic plot of the THLN fitted curve (red line) with pointwise 95% confidence band (red area), showing the data points (black dots) with their 95% confidence intervals (blue lines), Althabe et al. trial, hands-off treatment [1]

The goodness of fit of the THLN distribution to the data can be visualized in the lognormal probability plot of Fig. 2. The probabilities from the fitted lognormal distribution and the data points are on a straight line for values above 50 mL, thereby showing that the fit of the THLN distribution to the data is very good above 50 mL and providing evidence that the lognormal distribution is appropriate to the blood loss volume distribution. No outliers were detected. Only one treatment is shown in Fig. 2 for illustration purposes, because the two treatments had a very similar behaviour.

**Fig. 3:**
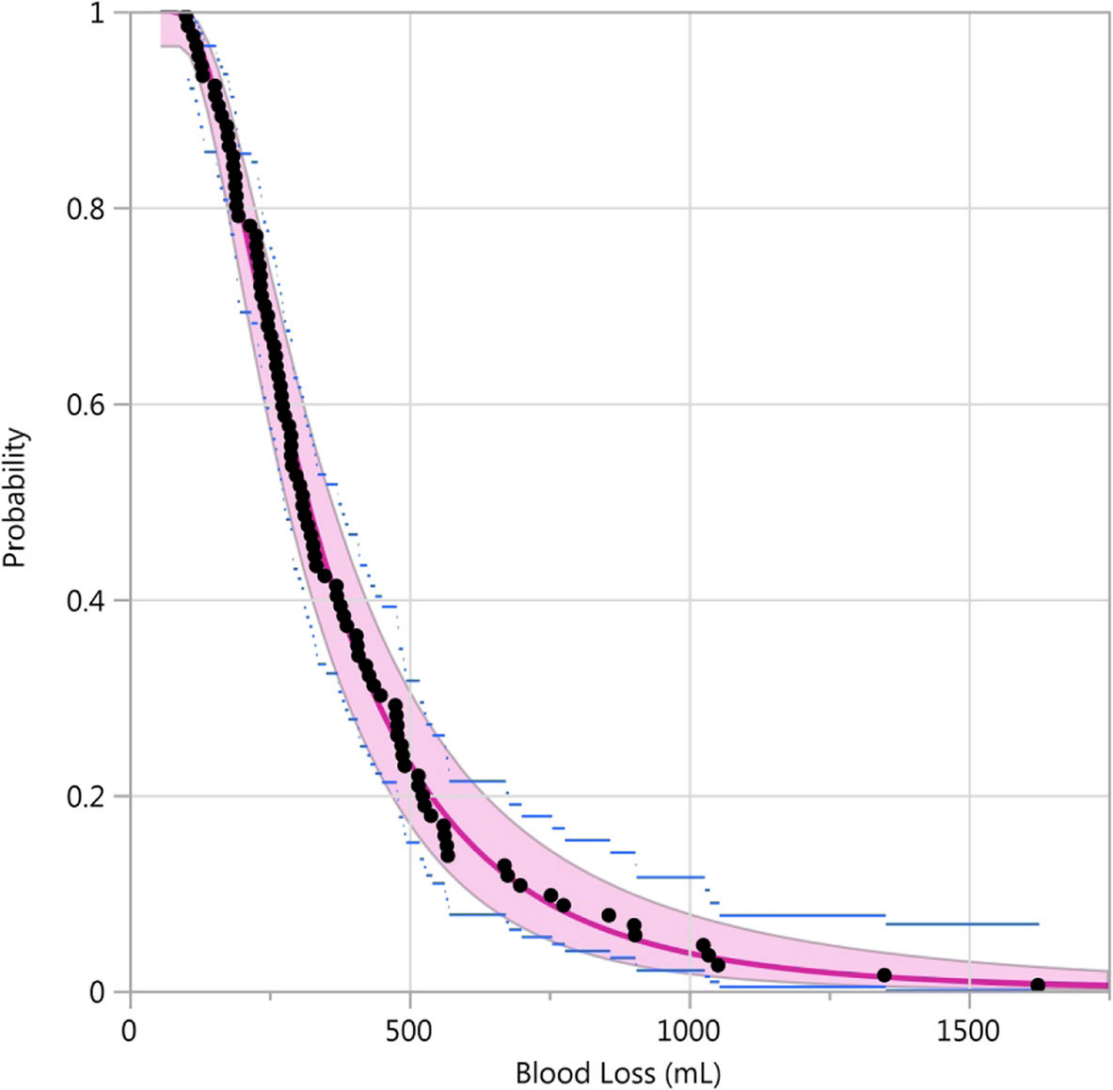
THLN fitted curve (red line) with pointwise 95% confidence band (red area), the empirical survival function (black dots) with the pointwise 95% confidence intervals (blue lines), Althabe et al. trial, hands-off treatment [1]

In Fig. 3 we show the survival function for the ‘hands-off’ treatment (*n* = 98), where the data points are represented by black dots and the pointwise 95% confidence intervals by blue lines. Figure 3 also shows the fit of a three-parameter lognormal distribution (THLN), in a superimposed red line, with 95% confidence band (red area). In Fig. 3 we can see, for example, that the probability of having 500 mL or more of blood loss is about 0.20. The lognormal distribution fits the data points very well. The 95% confidence intervals from the lognormal fit are narrower than the ones for the binomial estimates.

### Estimates of proportions and relative risk

Estimation of proportions using number of events divided by the total number in each treatment will be denoted the binomial approach. Estimation of proportions using the survival function at the relevant cut-off point will be denoted by the lognormal approach. We can see in Fig. 3 that the number of black dots above 1000 mL is 5, resulting in an estimated proportion by the binomial approach, of 5/98 = 0.051, or 5.1%, for the hands-off treatment. The estimate of the proportion of sPPH, based on the fitted survival function, is 0.038 (0.017 to 0.078). It is interesting to note that, of the five points in excess of 1000, three are close to the cut-off point. They could easily have slipped, by chance, to close values to the left of the cut-off, thereby sharply lowering the binomial estimate. Such a change would barely affect the lognormal estimate, since the fitted curve closely follows the entire cumulative empirical distribution and takes into account all the data.

Table 2 shows the estimated proportions of sPPH for the Althabe et al. trial by the binomial approach, as reported in the published trial results [1] and by the lognormal approach, per treatment group.

**Table 2:** Estimated proportions of sPPH for the Althabe et al. trial [1] by the binomial and by the lognormal approaches, per treatment group; treatments are hands-off and control cord traction (CCT)

Trial	Treatment	n/N	Proportion 95% CI	Width of the 95% CI	Width ratio lognormal vs binomial (%)
Binomial	Hands-off	5/98	0.051 (0.022 to 0.114)	0.092	–
	CCT	3/101	0.030 (0.010 to 0.084)	0.074	–
Lognormal	Hands-off	–	0.038 (0.017 to 0.078)	0.062	67
	CCT	–	0.033 (0.014 to 0.069)	0.055	75

As can be appreciated in Fig. 3, Table 2 also shows that the 95% confidence intervals for the lognormal approach are narrower than the ones for the binomial approach. For the hands-off treatment, for example, the width of the binomial estimate confidence interval is 0.092, whereas that of the lognormal estimate confidence interval is 0.062, about two thirds of the former. The 95% confidence intervals width ratio is 67% and 75% respectively for the hands-off treatment and for the controlled cord traction (CCT) treatment.

For the lognormal distribution, the relative risk, shown in Table 3, with the 95% confidence intervals, is estimated by the bootstrap technique, using 1000 bootstrap samples. Note that the confidence intervals for the RR are wide because this is a small trial that was designed to compare median blood loss as the main outcome.

**Table 3:** Relative risk with 95% confidence intervals for the binomial and lognormal approaches, and gain in precision, Althabe et al. trial [1]

Approach	RR (95% CI) CCT vs Hands-off	Ratio upper/lower limit of the 95% CI	Log scale width ratio lognormal vs binomial (%)
Binomial	0.58 (0.14 to 2.37)	16.9	–
Lognormal	0.86 (0.22 to 2.62)	11.9	70.4

From the comparison of the 95% confidence limits for both approaches, we obtained a log-scale width ratio of 70.4% for the lognormal approach in relation to the binomial approach, so that the gain in precision when using the lognormal instead of the binomial approach, is about 30%.

### Tests of hypothesis

The test of equality of sPPH proportions by the binomial method can be based on a 2 × 2 table, giving the Pearson chi-square = 0.591 with *p*-value = 0.4440.

The lognormal tests of hypothesis that the two distributions are the same is shown in Table 4. The comparison of the scale parameters shows that they are not significantly different, as the p-value equals 0.7381; therefore there is no evidence that the scale parameters of the two treatments are different. The location parameters are not significantly different either, as the p-value equals 0.1506. Hence we conclude that there is no evidence against the equality of the distributions.

**Table 4:** Results of lognormal tests of equality of distributions of the two treatments, Althabe et al. trial [1]

Models compared	Likelihood ratio Chi-square	DF	p-value
No effect vs. location	2.066	1	0.15
Location vs. location and scale	0.112	1	0.74

The significance level for the final test, comparing the location parameters, is about 0.15 for the lognormal approach. This can be compared to the significance level of the binomial test, 0.44. The lognormal approach seems more sensitive.

**Fig. 4:**
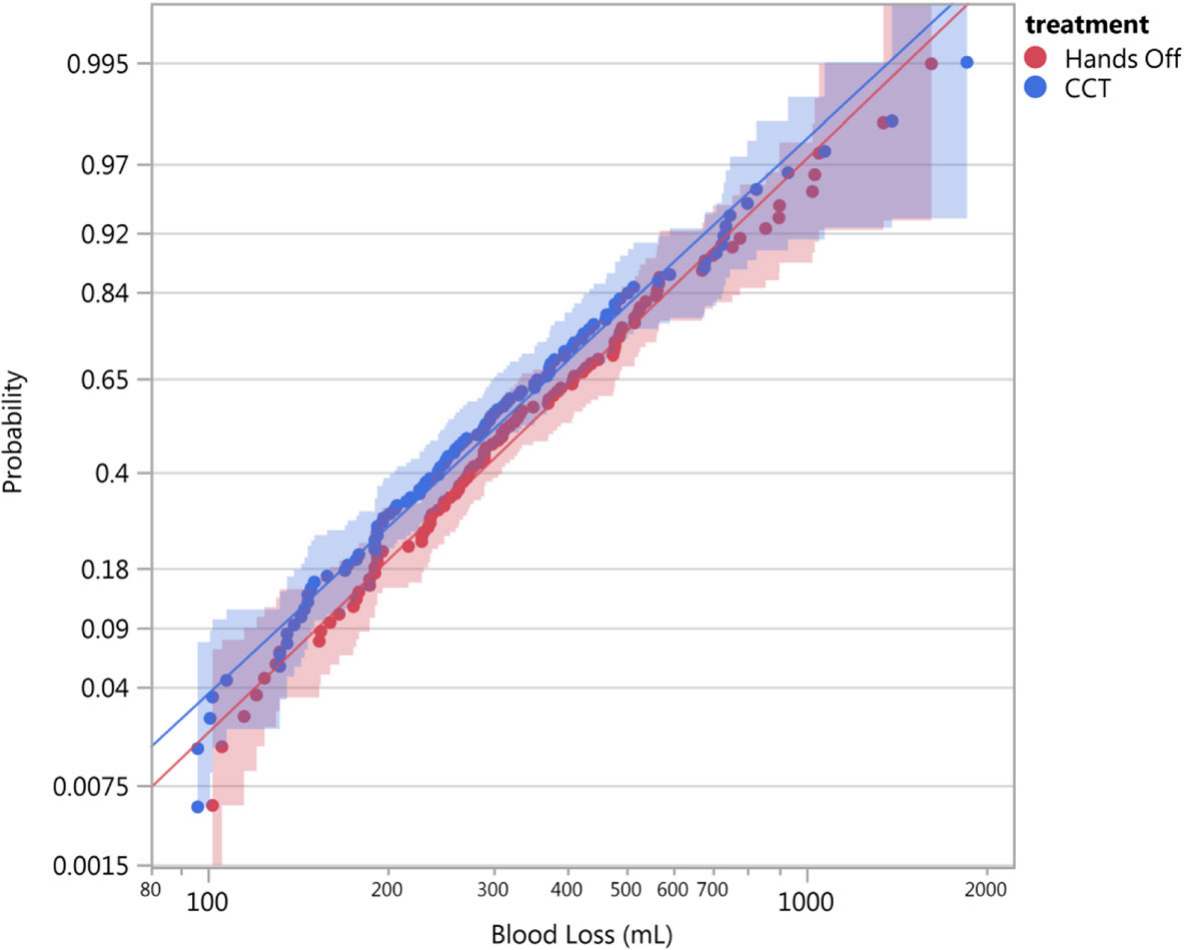
Distributions of blood loss for the two treatments, Hands-off (red) and CCT (blue), side by side on a lognormal probability plot, showing data points (dots), fitted lognormal lines (continued full lines), and 95% confidence bands (shaded areas), Althabe et al. trial [1]

Figure 4 shows the two distributions side by side on a lognormal probability plot. The joint confidence intervals overlap entirely, suggesting that the two distributions are very similar.

### Sample size: an example

We present as an example different scenarios of sample size calculation using the two approaches, the binomial and the lognormal one.

For the binomial approach, we assume that the proportion of sPPH is 0.015, 0.02 or 0.025 in the current treatment. We also assume that a new preventive therapy is considered worthwhile if the relative risk of sPPH of the new therapy with respect to the current one is no larger than 0.70 or 0.80. We calculated the sample size for the binomial response based on the likelihood ratio chi-square one-sided test for the relative risk statistic.

For the lognormal approach, we used the well known result that if a variable has a lognormal distribution, its logarithm has a normal distribution. Therefore, our sample size computations were based on the transformation of the volumes to their logarithms. For a scenario consisting of a given proportion of sPPH, say p, and a relative risk RR = p_2_/p_1_, we computed the corresponding risk of the competing treatment, p_2_. For the standard deviation on the log scale, we used the scale parameter obtained from the analysis of three clinical trials, that was in all cases close to s = 0.7. [4] The values of the mean m (on the log scale) can be readily computed for each value of p and for each scenario, from an equation derived from the proportion p of sPPH:

$$ \frac{\log (1000)-m}{s}={z}_{1-p} $$

where z_1-p_ is the (1-p) quantile (or (1-p)× 100% percentile) of the standard normal distribution, taking values p_1_ and p_2_ for a particular scenario. With the two computed means, derived from p_1_ and p_2_ using the equation above, and the (fixed) standard deviation, together with the power requirement, the computation of the sample size is straightforward, done as a comparison of means of the log-transformed variable blood loss. SAS PROC POWER with TWOSAMPLEMEANS was used for the computations.

The total sample sizes for the two approaches, for a power of 80%, in a one-sided 5% significance test, are shown in Table 5. The difference in required sample size is enormous, as expected, because the lognormal approach is using more of the information in the sample.

**Table 5:** Sample size required for the two different approaches, binomial and lognormal, under different scenarios, for a one-sided 5% significance test with 80% power for relative risk (RR)

Scenario	Assumed sPPH rate for control (%)	RR	Total sample size with the binomial approach	Total sample size with the lognormal approach
1	1.5	0.70	15,294	1178
2	1.5	0.80	36,522	3068
3	2.0	0.70	11,422	1080
4	2.0	0.80	27,266	2814
5	2.5	0.70	9098	1002
6	2.5	0.80	21,714	2618

## Discussion

We have illustrated how to apply an analysis technique for blood loss volume data based on the lognormal distribution, without categorizing this response variable, for a small trial [1], verifying first that the blood loss volume indeed follows a lognormal distribution. Using data from two large trials [9, 10], the same pattern was found, that we reported elsewhere [4]. For these two large trials, the fit was also very good. We have also fitted a lognormal distribution to blood loss data using the reported percentiles from an observational study [11]. In all cases, we have found empirical evidence that the blood loss volume has a lognormal distribution, and can be described by a variant of this family of distributions, the threshold three-parameter lognormal distribution [12]. A lognormal distribution with its specific parameters characterizes several physical and biological phenomena [16], and can be described by means of a physical model as a multiplicative sequence of losses.

Furthermore, the estimated location and scale parameters from all four of the studies analyzed were very similar [4]. The studies were conducted in different places and times, suggesting that the lognormal distribution fits postpartum blood loss data universally. The stability of the parameters found in the analysis of blood loss data across studies may well be a characteristic of postpartum blood loss that can be further explored.

The available analysis technique to compare proportions of an event of the type ‘blood loss above a certain cut-off point’ between treatments or interventions to prevent this event, has been to estimate the two binomial proportions of sPPH. The categorization of blood loss volume in two categories entails loss of information contained in objectively measured weight or volume data, with a resulting loss of power in tests of hypothesis and a decrease in the precision of the estimates of proportions and relative risks [8]. This fact, together with the low prevalence of rare events when the cut-off point is a high value of blood loss volume, for example 1000 mL, results in very large size of trials needed to compare this event between treatments or interventions.

We proposed a lognormal approach of analysis of postpartum haemorrhage trials aiming to compare events of the type ‘blood loss greater than a certain cutoff point’ between treatments. [4] We illustrate here this approach, consisting of fitting a lognormal type of distribution to blood loss data, which involves estimating the parameters that define the distribution. Once the distribution function of blood loss volume, or its complement, the survival function, is defined by its parameters, the proportion of sPPH, for example, is just the survival distribution at the point 1000. To compare treatments using relative risk, we estimated its confidence interval with bootstrap techniques.

An application of using the lognormal model for the distribution of the blood loss volume is a substantial reduction of sample size of clinical trials, while keeping the statistical power and precision requirements. Using the lognormal approach that we propose, based on fitting a lognormal distribution to the postpartum blood loss data, the objectives of a trial can be attained with smaller sample sizes and reduced cost, through an improvement in the efficiency of the estimation methods. With the lognormal approach, there is a trade-off between the simplicity offered by the binomial approach and the possibility of reducing the size of a trial.

Similar methods described in this paper can be used with other variables having the lognormal distribution, like blood pressure [3] and estimation of hypertension. The reason why this approach has not been used in the past, is that it is computer intensive. Nowadays, with improvement in computer power, this is not a problem, although the complexity of fitting a lognormal distribution and calculating relative risk’s confidence intervals by bootstrapping requires statistical expertise.

As an additional bonus to the use of the lognormal approach based on fitting a lognormal distribution to blood loss data, it is possible to test other hypotheses of interest, such as the equality of medians or any other percentile, or even compare the entire distributions between two treatments or interventions.

## Conclusions

We illustrated how a lognormal approach based on fitting a lognormal distribution to the data can be applied to measured blood loss volume data of a trial. We found that the precision of the estimates of proportions of the event ‘blood loss greater than 1000 mL’ and its comparison between treatments improved compared to the standard methods based on dichotomizing the blood loss variable. We also illustrate how the lognormal approach can be used to compare the distribution parameters for two treatments. When analyzing data using this lognormal approach, sample size of trials can be reduced.
